# Development of a Universal Multi-Epitope Vaccine Candidate against *Streptococcus suis* Infections Using Immunoinformatics Approaches

**DOI:** 10.3390/vetsci10060383

**Published:** 2023-05-31

**Authors:** Yumin Zhang, Guoqing Zhao, Yangjing Xiong, Feiyu Li, Yifan Chen, Yuqiang Cheng, Jingjiao Ma, Henan Wang, Yaxian Yan, Zhaofei Wang, Jianhe Sun

**Affiliations:** Shanghai Key Laboratory of Veterinary Biotechnology, School of Agriculture and Biology, Shanghai Jiao Tong University, Shanghai 201100, China

**Keywords:** *Streptococcus suis*, multi-epitope vaccine, immunoinformatics, epitope prediction, molecular docking

## Abstract

**Simple Summary:**

*Streptococcus suis* has been receiving increasing attention due to its involvement in severe human infections worldwide. However, there is currently no licensed vaccine against *S. suis*. Therefore, vaccine development is very essential to eliminate the threat of this human pathogen. Herein, we contrived a multi-epitope subunit vaccine against *S. suis* isolates of multiple serotypes using immunoinformatics. Further, the characteristics of the designed vaccine were evaluated using several analyses, including antigenicity, allergenicity, toxicity, and tertiary structure analyses and molecular docking; the results indicated that it could be an effective universal vaccine against *S. suis* infections.

**Abstract:**

*Streptococcus suis* is a significant zoonotic pathogen that is a great threat not only to the swine industry but also to human health, causing arthritis, meningitis, and even streptococcal toxic shock-like syndrome. Owing to its many serotypes and high geographic variability, an efficacious cross-protective *S. suis* vaccine is not readily available. Therefore, this study aimed to design a universal multi-epitope vaccine (MVHP6) that involved three highly immunogenic proteins of *S. suis*, namely, the surface antigen containing a glycosaminoglycan binding domain (HP0197), endopeptidase (PepO), and 6-phosphogluconate dehydrogenase (6PGD). Forecasted T-cell and B-cell epitopes with high antigenic properties and a suitable adjuvant were linked to construct a multi-epitope vaccine. In silico analysis showed that the selected epitopes were conserved in highly susceptible serotypes for humans. Thereafter, we evaluated the different parameters of MVHP6 and showed that MVHP6 was highly antigenic, non-toxic, and non-allergenic. To verify whether the vaccine could display appropriate epitopes and maintain high stability, the MVHP6 tertiary structure was modeled, refined, and validated. Molecular docking studies revealed a strong binding interaction between the vaccine and the toll-like receptor (TLR4), whereas molecular dynamics simulations demonstrated the vaccine’s compatibility, binding stability, and structural compactness. Moreover, the in silico analysis showed that MVHP6 could evoke strong immune responses and enable worldwide population coverage. Moreover, MVHP6 was cloned into the pET28a (+) vector in silico to ensure the credibility, validation, and proper expression of the vaccine construct. The findings suggested that the proposed multi-epitope vaccine can provide cross-protection against *S. suis* infections.

## 1. Introduction

*Streptococcus suis* (*S. suis*), an encapsulated, potentially pathogenic, gram-positive bacterium, is one of the resident flora of pig tonsils [1]. It possesses high phenotypic heterogeneity with at least 29 serotypes, described based on the immunogenicity of capsular polysaccharide (CPS), and 1003 sequence types, described based on the seven housekeeping genes. *S. suis* is widespread across pig farms worldwide and responsible for systemic infections, including septicemia and meningitis, and even sudden death, thereby causing considerable financial losses to the livestock industry [2]. Moreover, *S. suis* is a newly emerging zoonotic agent capable of causing conditions ranging from mild illness to severe health problems, including streptococcal toxic-shock-like syndrome in humans [3,4]. The occurrences of two major *S. suis* infections, resulting in high mortality rates among humans in the provinces of Jiangsu and Sichuan, China, have attracted great attention [5,6]. Currently, *S. suis* infections in humans are also frequently reported in Southeast Asia [7]. Intriguingly, among the various serotypes, the serotypes 2, 4, 5, 7, 9, 16, 24, and 31 are potentially transmitted from pigs to humans [7].

Antibiotic overuse by farmers has increased antimicrobial resistance in *S. suis*, posing a challenge to clinical medication for infected persons [8,9]. Therefore, owing to the presence of various serotypes as well as the escalating drug resistance in *S. suis*, it has become necessary to develop a universal vaccine that can provide cross-protection against *S. suis* infections for humans, particularly certain populations (such as veterinarians and butchers) who are at high risk of exposure to diseased pigs or pork products [10].

There are currently no universally efficacious commercial vaccines against *S. suis* infections. *S. suis* outbreak prevention in a particular farm is mainly achieved through autogenous (bacterins) vaccines; however, vaccine cross-protection between different strains is limited and data regarding vaccine safety are lacking [10]. In the past two decades, various immunogenic proteins that are highly conserved across different serotypes have been shown to elicit immune responses in animal models [11,12,13,14]. The development of a vaccine composed of multiple protein antigens is probably an attractive strategy to control infections caused by a broad range of serotypes through the targeting of several important virulence factors [15,16]. Recently, *Salmonella enterica* serovar *Choleraesuis* vectors delivering conserved surface proteins have provided cross-protection against the *S. suis* serotypes 2, 7, 9, and ½ in mice [17].

With the development of modern vaccinology and immunoinformatics, the use of the multi-epitope vaccine is a good strategy to combine multiple proteins, owing to the vaccine’s cost-effectiveness, safety, potential for rational epitope design to amplify efficacy, and ability to cover a broader spectrum of serotypes. At present, the multiple-epitope vaccine approach has been shown to be promising against- SARS-CoV-2 [18], coxsackievirus B [17], *Toxoplasma gondii* [19], *Mycobacterium tuberculosis* [20], and *Staphylococcus aureus* [21]. Herein, we developed a universal multi-epitope vaccine candidate against *S. suis* based on multiple conserved proteins using advanced bioinformatics tools. To this end, three immunogenic proteins of *S. suis*, namely, HP0197 [22], PepO [23], and 6PGD [24], which are surface proteins and highly conserved in *S suis*, were employed for epitope prediction. Notably, immunogenic proteins were selected as their ability to confer varying levels of immune protection in both mice and pigs has been confirmed using experimental data.

## 2. Materials and Methods

### 2.1. Retrieval of Protein Sequences

To conduct this project, HP0197, PepO, and 6PGD were employed as candidate antigenic proteins to predict epitopes, which are the major components of the multi-epitope vaccine. The protein sequences were retrieved from NCBI with Genbank ID, ABP91355.1 (HP0197), ABP89124.1 (PepO), and ABP92903.1 (6PGD). The VaxiJen 2.0 server was employed to predict the antigenicity scores of the candidate proteins [25].

### 2.2. Prediction of B-Cell (BCL) Epitopes

In this project, the ABCpred server was used to predict specific B-cell epitopes of three candidate proteins [26]. The score threshold and the amino acid length were fixed at 0.85 and 16, respectively, in this work. In addition, the antigenicity of the predicted B-cell epitopes was further tested by the VaxiJen v2.0 server. The epitopes with an antigenicity threshold above 0.85 were selected. The allergenicity and toxicity predictions of epitopes were performed through the Allertop 2.0 server [27] and Toxinpred server [28], respectively.

### 2.3. Predictions of Cytotoxic T-Lymphocyte (CTL) Epitopes

The Immune Epitope Database (IEDB) webserver was utilized to predict CTL epitopes [29]. The IEDB MHC-I immunogenicity tool was utilized for the identification of immunogenicity [30]. The epitopes with an immunogenicity of more than 0.3 were readied for further analysis.

### 2.4. Predictions of Helper T-Lymphocyte (HTL) Epitopes

In this study, the HTL epitopes were predicted using IEDB MHC-II [31]. We used a seven-allele HLA reference set and IEBD recommended 2.22 predicted method to predict HTL epitopes. Further, the epitopes provided in the previous step were evaluated and screened based on their antigenicity and cytokine-inducing abilities through the IFN epitope [32], IL4pred [33], and IL10pred [34] servers, respectively.

### 2.5. Predicted Epitopes Conservation Analysis

To explore the conservation of selected epitopes, the analysis was performed using the local blast software. Firstly, 30 *S. suis* strains, including 3–5 strains of *S. suis* from each serotype potentially infecting humans, were retrieved from Genbank in the FASTA format and established a nucleic acid database. Then, the amino acid sequences of the selected epitopes were then subjected to tBLASTn analysis to search for similarity with default parameters. Subsequently, BacWGSTdb (http://bacdb.cn/BacWGSTdb/index.php, accessed on 26 November 2022) was used to conduct a phylogenetic tree of the selected 30 strains based on SNP strategy, and their MLST types were counted. The phylogenetic tree was landscaped through Chiplot (https://www.chiplot.online/tvbot.html, accessed on 7 December 2022).

### 2.6. Multiple-Epitope Vaccine Designing and Processing

The epitopes were fused by linkers, AAY, KK, and GPGPG, which are commonly used in vaccine construction. The order of the epitopes was linked according to the hydropathicity, which could be calculated by the ExPasy [35]. The N-terminus of this epitope fusion sequence was adjuvanted with 50S ribosomal protein L7/L12 (NCBI ID: P9WHE3), a toll-like receptor 4 (TLR4) agonist, via the EAAAK linker.

### 2.7. Prediction, Refinement, and Validation of the Tertiary Structure

The I-TASSER server was utilized to model the initial 3D structure of the designed vaccine [36]. Further, the 3D model with the highest score from I-TASSER was submitted to the GalaxyRefine server to refine the initial 3D structure [37]. In addition, the ProSA-web, ERRAT and PROCHECK servers were employed to assess and validate the quality of the modeled or refined 3D structures of the vaccine [38,39,40].

### 2.8. Population Coverage

Based on the Allele Frequency Net Database, the IEDB population coverage analysis tool measures the population coverage of corresponding epitopes by targeted HLA allele genotypic frequencies [41]. Hence, to check the population coverage of the vaccine designed in this study, shortlisted epitopes, along with their corresponding identified HLA allele types, were submitted to the tool.

### 2.9. Molecular Docking and Molecular Dynamics (MD) Simulation

To check the binding affinity of the designed vaccines to immune receptors, in this study we performed a molecular docking analysis between the vaccine construct and several immune receptors [42], including Toll-like receptors such as TLR2 (PDB: 2Z7X) and TLR4 (PDB: 4G8A), and major histocompatibility complex molecules such as MHC-I (PDB: 4U6Y) and MHC-II (PBD: 5JLZ). The PDBsum server was utilized for vaccine–receptor contact analysis [43], while structural illustrations were visualized through the PyMOL. Moreover, to evaluate the stability of the vaccine–receptor binding complex, the iMOD server was employed to perform a molecular dynamics analysis [44].

### 2.10. Immune Response Simulation

To assess the vaccine’s potential immunological response to the vaccine, we submitted the entire sequence of the vaccine construct MVHP6 to the C-IMMSIM server to predict its ability to provoke a possible immune response [45]. As described previously [46], the time steps for the three injections in the in silico administration were set as 1, 84 and 168, respectively, with one time step being equal to 8 h in real life. The remaining criteria were set to the default for the prediction.

### 2.11. Codon Optimization and In Silico Cloning

To achieve the high-quality expression of design vaccine proteins in the prokaryotic expression system *E. coli* K12, we submitted the amino acid sequence to the JCAT sever for reverse translation and codon optimization to obtain an improved DNA sequence [47]. Subsequently, the vaccine’s DNA sequence was subjected to computational cloning with the pET28a (+) expression vector using Snapgene software (version 5.1.6).

## 3. Results

### 3.1. Prediction and Analysis of BCL Epitopes

BCL epitopes were predicted using the ABCPred tool based on their high binding affinity to BCL receptors. Epitopes with the highest binding and antigenicity scores among all potential epitopes are listed in Table S1. Our results show that the numbers of shortlisted epitopes for HP0197, PepO, and 6PGD proteins were 7, 2, and 10, respectively. The epitope from the 6PGD protein had the highest antigenicity score (1.4914), whereas the epitope from the PepO protein had the lowest antigenicity score (0.8605) (Table S1). Based on the antigenicity score, the top two BCL epitopes from each protein were considered for the next vaccine construction (Table 1).

### 3.2. Prediction of CTL Epitopes

The CTL epitopes were predicted using the IEDB v2.24 webserver. All predicted CTL epitopes with binding affinity for major histocompatibility complex I (MHC I) were subsequently submitted to a series of servers to obtain the desired peptide-containing epitopes that are non-toxic, non-allergenic, and have antigenicity and immunogenicity scores of higher than 0.85 and 0.3, respectively (Table S2). One CTL epitope from each protein was selected separately for subsequent vaccine construction based on its high antigenicity (Table 1).

### 3.3. Prediction of HTL Epitopes

The HTL epitopes of three candidate antigen proteins of *S. suis*, namely, HP0197, PepO, and 6PGD, are predicted and presented in Table S3, which shows a comprehensive list of the HTL epitopes based on binding affinity, non-toxicity, non-allergenicity, antigenicity, and the ability to induce IFNγ, IL4, and IL10. Overall, three HTL epitopes (one HTL epitope from each protein with positive IFNγ scores and the potential to induce IL4 and IL10) were selected for vaccine construction (Table 1).

### 3.4. Analysis of Epitope Conservation

**Figure 1:**
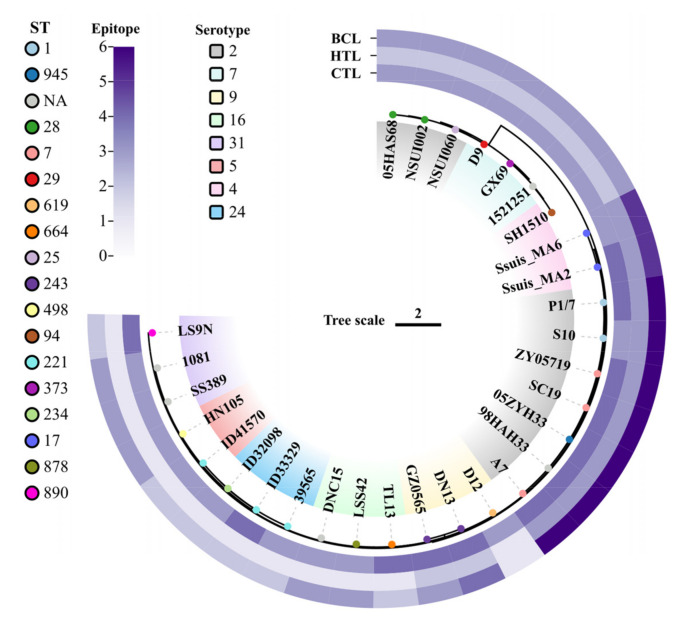
Conservative analysis of predicted epitopes against strains of *S. suis* infecting humans. The phylogenetic tree was constructed based on SNPs from the genomes of 30 human-infecting *S. suis* strains. The different base colors of the strain names show the different serotypes. The colored circles represent the MLST types of the different strains. The outer layer (from the inner to the outer part), shows the conservation of cytotoxic T-lymphocyte, helper T lymphocyte, and B-cell epitopes, respectively. Within each category, the distribution of exact matches varies from 1 to 6 across strains.

To verify the ubiquity of the 12 predicted antigenic peptides in *S. suis*, the peptides were submitted to local BLAST based on a database containing 30 *S. suis* genomes covering serotypes 2, 4, 5, 7, 9, 16, 24, and 31. For each epitope, strains with 100% amino acid sequence matching were collected. The phylogenetic tree was built using the strains’ SNPs, as well as serotype, MLST, and epitope conservation. CTL, HTL, and BCL epitopes were covered in all 30 *S. suis* strains (Figure 1). Furthermore, at least 2/3 CTL epitopes coexisted in each *S. suis* strain, and more than 3 CTL epitopes were present in several serotypes of *S. suis* strains (i.e., serotypes 2, 4, 9, 16, 24, and 31), indicating that the epitope sequence appeared more than once in the genomes of *S. suis* strains. These results indicated that the selected 12 epitopes could cover highly susceptible serotypes in humans, suggesting the potential of the final multiple-epitope vaccine to protect humans from infections caused by various serotypes of *S. suis*.

### 3.5. Estimated Population Coverage

**Figure 2:**
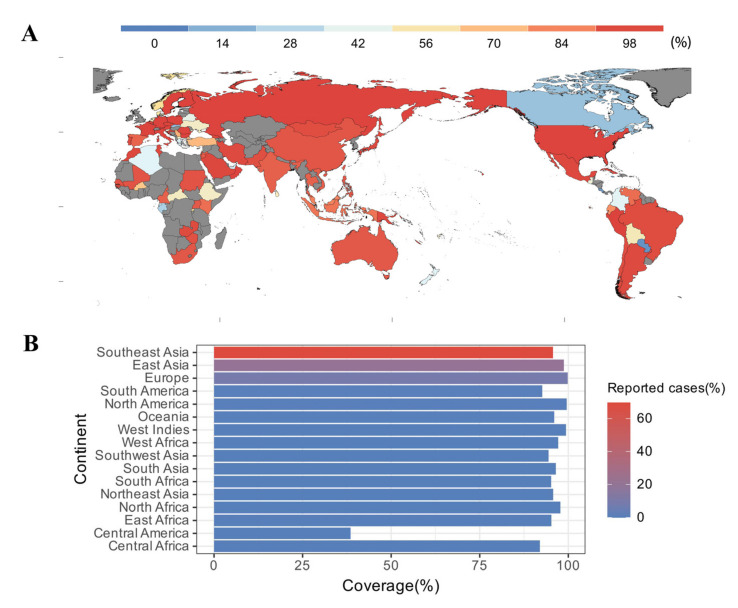
Population coverage analysis. (**A**) World map indicating population coverage in different regions, predicted based on the selected epitopes; countries shown in gray indicate unavailability of data. (**B**) Population coverage and condition of reported cases in different regions. Reported cases are derived from the reference [48].

To predict population coverage, the selected epitopes and their respective HLA alleles were submitted to the IEDB server. The results showed that the epitopes were predicted to cover 99.19% of the world population. For countries with a developed pig industry, such as China, Vietnam, and Thailand, the population coverage was as high as 95.63%, 93.3%, and 92.83%, respectively (Figure 2A). Meanwhile, in regions where human cases of *S. suis* infection were frequently reported, such as Southeast Asia (69.73%), East Asia (20.46%), and Europe (8.53%), the population coverage could reach 95.68%, 98.75%, and 99.83%, respectively (Figure 2B).

### 3.6. Construction of the Multi-Epitope Vaccine (MVHP6)

**Figure 3:**
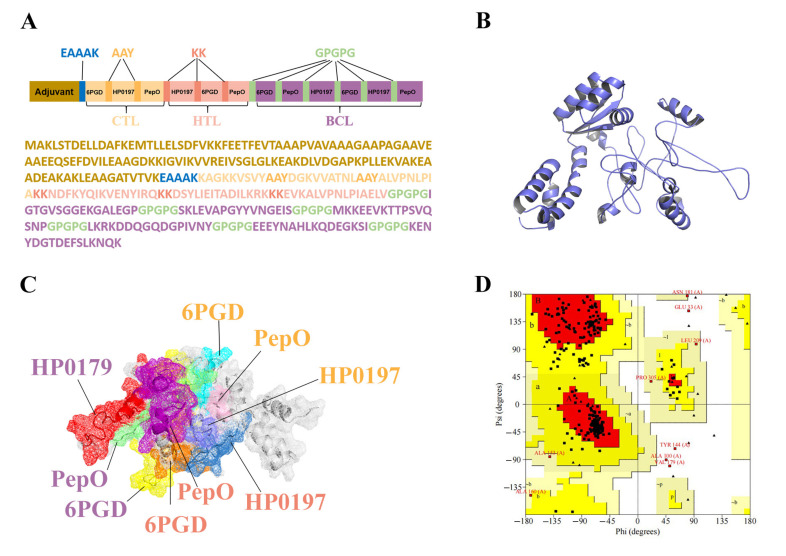
Analysis of the finalized multi-epitope vaccine (MVHP6) construct. (**A**) Schematic representation of the sequence and epitope alignment concerning the MVHP6 construct. (**B**) Predicted and refined 3D structure of MVHP6. (**C**) Presentation of epitopes in the 3D structure of MVHP6. Different epitopes are indicated using different colors; white areas represent adjuvants and linkers. The labeled proteins have CTL epitopes in orange, HTL epitopes in pink, and BCL epitopes in purple. (**D**) Ramachandran diagram of the refined MVHP6 construct. Red region: most favorable [A, B, L]; yellow region: additional allowances [a,b,l,p]; pale yellow area: generous allowances [~a,~b,~l,~p].

To chimerize multiple peptides, the 3 CTL, 3 HTL, and 6 BCL epitopes were separated by AAY, KK, and GPGPG linkers, respectively, as shown in Figure 3A. To optimize vaccine solubility, the epitopes in each category were rearranged so that the hydrophilic epitopes were at the ends of the chimeric proteins. Moreover, the EAAAK linker was employed to join the first epitope of CTL and the adjuvant sequence, 50S ribosomal protein L7/L12 (130 amino acids) (Figure 3A). The final MVHP6 construct is composed of 345 amino acid residues. Importantly, MVHP6 exhibited non-toxicity and non-allergenicity, as well as a possible antigen with a high antigenicity score (0.87).

### 3.7. Tertiary Structure Prediction, Refinement, and Validation of MVHP6

Upon providing the vaccine sequence, the I-TASSER server generated five predicted 3D structures of MVHP6, and the model 3, with the highest C-score of −3.54, was selected for further study. To further improve the 3D structures of the vaccine, the GalaxyWEB server was used to obtain the final 3D structure of MVHP6 (Figure 3B). The epitopes shown in different colors were found on the surface of the constructed vaccine (Figure 3C). The improved model was evaluated and showed an increase in the Z score from −6.36 to −7.01 according to ProSA-web (Figure S1). Moreover, Ramachandran analysis verified 82.7%, 14.4%, and 1.4% of residues in the red region (most favorable), yellow region (additional allowances), and pale yellow area (generous allowances), respectively. Approximately 1.4% of the vaccine’s residues were found in forbidden locations (highlighted in white) (Figure 3D). The vaccine 3D structure has an overall quality score of 79.1139, evaluated by the ERRAT server, indicating that the overall model quality is satisfactory (Figure S2).

### 3.8. Molecular Docking of the Constructed Vaccine with the Human Toll-like Receptor, TLR4

**Figure 4:**
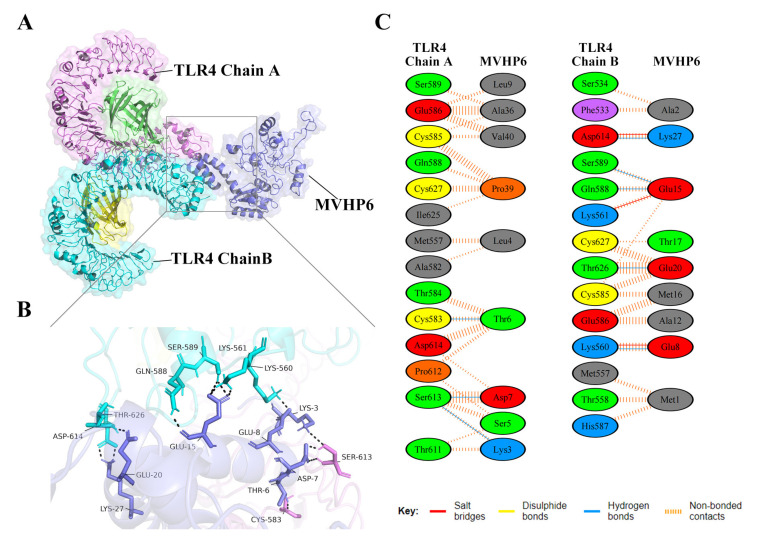
Molecular docking of the vaccine with TLR4. (**A**) Diagram of the docking pattern of the multi-epitope vaccine (MVHP6)-TLR4 complex. (**B**) Diagram of the docking conformation and hydrogen bonding interactions of MVHP6 (shown in purple) with TLR4 chain A (shown in pink) and chain B (shown in bright blue); the black dashed lines refer to the hydrogen bonds. (**C**) Residues of the interaction between docked MVHP6 and TLR4.

Vaccines should have a high affinity for host immune receptors such as the Toll-like receptors and MHC molecules to induce an optimal immune response. Herein, molecular docking analysis using ClusPro 2.0 revealed that MVHP6 had a strong binding affinity to TLR4 (Figure 4A). The vaccine-TLR4 complexes showing H-bond interactions were LYS3-SER613, THR6-CYS583, ASP7-SER613, GLU8-LYS560, CLU15-LYS561, CLU15-SER589, CLU15-CLN588, CLU20-THR626, and LYS27-ASP614. In addition to hydrogen bonding, the PDBsum server was used to comprehensively understand the binding residues between the docked complexes. The results showed 3 salt bridges, 142 non-bonded contacts, and an absence of disulfide bonds between the interacting atoms of the MVHP6-TLR4 docked complex (Figure 4B,C). Furthermore, MVHP6 showed considerable interactions with TLR2, MHC-I, and MHC-II (Figures S3–S5).

### 3.9. Molecular Dynamics (MD) Simulation

**Figure 5:**
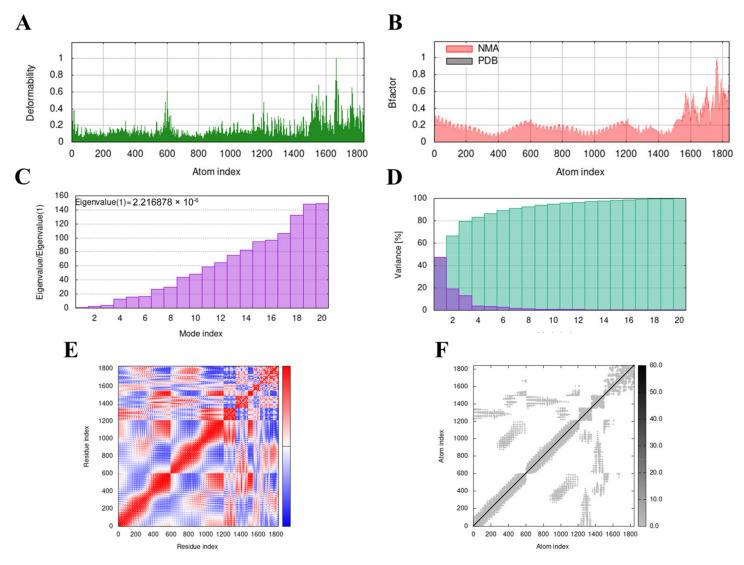
Molecular dynamics simulation of the MVHP6-TLR4 complex. (**A**) Deformability map of the docking complex. (**B**) NMA calculation of the B factor. (**C**) Eigenvalues of the docked complex. (**D**) NMA variance. (**E**) Covariance matrix plot of the atomic pairs of amino acid residues of the docked complex. (**F**) Connection spring plot of the elastic network model of the docked complex.

The MD simulation of the MVHP6-TLR4 docking complex was performed using the iMODS server. The deformability builds up the independent distortion of each residue, portrayed by the method of chain hinges (Figure 5A). Figure 5B shows the deformability plot of both complexes, where the peaks indicate the non-rigid regions of the complexes. The Eigenvalue of the MVHP6-TLR4 docking complexes was 2.216878 × 10^−6^ (Figure 5C). Additionally, the variance matrix graph of the residues is inversely related to the eigenvalue, showing the individual (red) and cumulative (green) variances (Figure 5D). The covariance matrix signifies the coupling between pairs of residues, representing the correlation experience: red represents correlated, white represents uncorrelated, and blue shows anti-correlated motions (Figure 5E). Figure 5F shows the elastic network of the complexes, where dots indicate one spring and a gray area indicates stiffer springs. These results suggest stable binding interactions with compact conformation and minor fluctuations in the vaccine—TLR4 complex.

### 3.10. Immunogenicity Evaluation of the Vaccine

**Figure 6:**
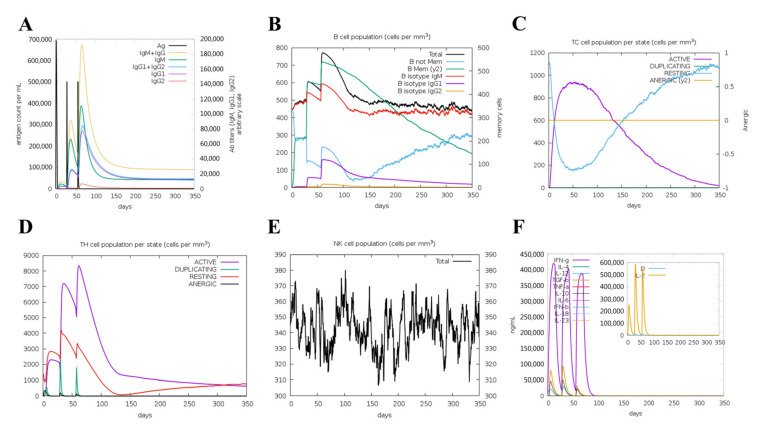
The immune response of humans after vaccination using the multi-epitope vaccine. (**A**) Increases in antibodies after vaccination. (**B**) Increased numbers of B cells and levels of memory immunoglobulins following vaccination. Increased numbers of T lymphocytes (**C**) and helper T lymphocytes (**D**) after vaccination, which remained at high levels throughout the exposure time. (**E**) Behavior of the population of natural killer cells. (**F**) Induction of cytokines and interleukins.

The simulated immune response revealed that antibody levels increased progressively with the number of immunizations (Figure 6A). Figure 6B,C shows that the vaccine was designed to accumulate increased B-cell and T-cell populations. A considerable increase in Th1 concentration was observed after each dose. Additionally, a significant increase in macrophage numbers was observed following the injection (Figure 6D,E). Observably, various cytokines exhibited a robust increase in levels after injection (Figure 6F). Overall, these results suggested that MVHP6 could be effective in eliciting a robust immune response in silico.

### 3.11. In Silico Cloning of the Vaccine Candidate

**Figure 7:**
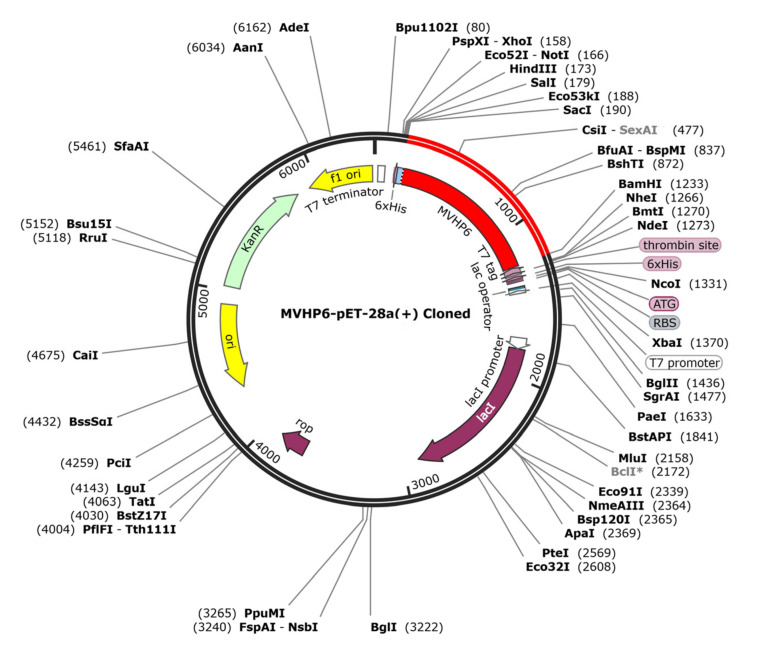
The in silico cloning of the optimized vaccine (shown in red) into the pET28a (+) expression vector.

The JCAT tool analysis revealed that the optimized sequence of MVHP6 had a codon adaptation index value of 1 and a GC content of 48.89%, indicating a high probability of the prokaryotic expression of the vaccine construct. We selected the *BamHI* and *ScaI* restriction sites and then successfully inserted the optimized sequence into the pET28a (+) vector, resulting in a 6404 bp cloned vector (Figure 7).

## 4. Discussion

*S. suis* can cause serious food safety problems and is harmful to public health, especially in countries with developed pig industries. With the continuous increase in *S. suis* resistance, a universal vaccine that can protect humans against various serotypes of *S. suis* is urgently needed. Currently, vaccine research on *Streptococcus pneumoniae* has focused on the development of polysaccharide conjugate-based vaccines for humans, which provides opportunities for multivalent vaccines against *S. suis* infections. However, there are still limitations to the development of multivalent polysaccharide conjugate-based vaccines, despite the success of the *S. suis* serotype 2 (SS2) vaccine construction. In order to address the challenges posed by diverse serotypes and geographic prevalence, we developed a multi-epitope vaccine by combining epitopes from three highly conserved antigenic proteins (6PGD, HP0197, and PepO) using in-silico-based predictions. We also evaluated its potential as a universal vaccine to trigger serotype-independent immunity at a low cost.

To ensure the cross-protection of a multi-epitope vaccine, it is important to select antigenic proteins that are conserved across all *S. suis* strains, regardless of serotype. BLASTP analysis showed that 6PGD, HP0197, and PepO were conserved in whole sequenced *S. suis* strains published in the NCBI, which is very promising for predicting epitopes with high conservation across multiple serotypes. Moreover, it is equally important to select candidate proteins that have been experimentally proven to provoke an immune response in the host, which could increase the likelihood of the epitope being successfully recognized by the host.

HP0197 is important in CPS synthesis, bacterial adhesion via HP0197-GAG-binding interaction, and virulence [49,50]. Importantly, HP0197 is a surface protective antigen that offers significant protection against lethal doses of SS2 in mice and pigs [22]. Moreover, HP0197 induces high titers of IgG antibodies, which provides complete protection in a passive immunized mouse model [22]. Previously, specific antibodies to HP0197 were detected in all convalescent sera of pigs and in sera from patients, indicating that HP0197 is an excellent candidate antigen against SS2 infection [51].

PepO, an important virulence factor, can cleave C3b via activated plasmin, enabling *S. suis* to evade innate immunity [52] and facilitate *S. suis* adherence to HBMEC cells by binding to fibronectin, thereby helping *S. suis* to cross the blood–brain barrier and cause meningitis [53]. Recently, it was shown that PepO elicited a strong immune response and provided complete protection in mice and partial protection in piglets when challenged with lethal doses of SS2 [23]. Furthermore, anti-rPepO serum provided good passive protection against SS2 infection in a mouse model [23]. Similarly, the PepO + PsaA combination vaccine significantly reduced *S. pneumoniae* colonization in the nasopharynx and lungs. Interestingly, *S. pneumoniae* PepO induced autophagy in a TLR2/TLR4-dependent manner, promoting macrophage phagocytosis and bactericidal activity. For example, *S. pneumoniae* PepO protected C57/BL6 mice from lung infections and reduced *S. pneumoniae* and *Pseudomonas aeruginosa* loads [54]. Notably, PepO has been proposed as a TLR agonist adjuvant candidate to enhance vaccine immunogenicity [55].

6PGD, a rate-limiting enzyme of the pentose phosphate cycle, is normally found in the cytoplasm. Importantly, 6PGD is identified as a surface protein involved in the adhesion and colonization of bacteria such as *S. pneumoniae* [56], *S. suis* [57], and *Haemophilus parvum* [58]. Recently, it was shown that *S. suis* r6PGD triggers high titers of specific antibodies, protecting 50% of piglets [24] and 80% of mice [57]. Additionally, mice immunized with live attenuated *Salmonella cholerae* vaccines carrying 6PGD from SS2 achieved a more than 90% survival rate when challenged with 10 or 20 × LD_50_ SS2 [59].

Epitopes predicted from candidate antigens were selected from T and B cell epitopes based on immunogenicity and antigenicity. A conservativeness analysis revealed that the selected epitopes could cover the reported serotypes that infect humans, owing to the candidate proteins being broadly distributed in the genome with high conservation. Vaccine constructs were designed by combining CTL, HTL, and BCL epitopes using suitable linkers that are important in increasing expression, stabilization, and folding. Additionally, vaccines based on multiple epitopes require adjuvant coupling as they lack immunogenicity when used alone. Indeed, several bacterial proteins, such as SLY, c-di-GMP, and PLY [60,61,62], trigger significant immune responses. Specific fragments from bacterial proteins are also frequently added as protein adjuvants to the amino acid sequence of subunit vaccines, such as β-defensin, ribosomal proteins, ESAT6, and HBHA [63,64]. Herein, the 50S ribosomal protein L7/L12 was selected and linked to CTL via a linker, an excellent adjuvant from *Mycobacterium tuberculosis* responsible for activating the polarization of CD4+ and CD8+ T cells and inducing T-cell-mediated cytotoxicity [65]. Based on the above, our vaccine construct, MVHP6, strongly interacted with TLR4 and proficiently bound with MHC I and MHC II, implying that the epitopes from the vaccine could be delivered efficiently to the host. Moreover, an MD simulation analysis of molecular docking showed that MVHP6-TLR4 requires a minimum energy for stable binding. Naturally, immune response simulations revealed that the final MVHP6 construct could induce the anticipated strong primary, secondary, and tertiary immune responses in vivo, providing an assurance of vaccination effectiveness prior to animal testing. These findings suggested that multi-epitope vaccine development is reasonable and appropriate, paving the way for controlling *S. suis* infections in the future.

## 5. Conclusions

This study introduces a universal multi-epitope vaccine candidate that could provide protection to humans against various serotypes of *S. suis* infections. Evaluation with the most reliable tools showed that the novel vaccine has desirable immunodominant properties and high population coverage. Therefore, we believe that multi-epitope vaccines could be an appropriate strategy for the development of serotype-independent vaccines.

## Figures and Tables

**Table 1:** The selected epitopes for the multi-epitope vaccine.

Epitope	Protein	Sequence	Antigenicity	Hydropathicity
BCL	HP0197	MKKEEVKTTPSVQSNP	1.1755	−1.35
		EEEYNAHLKQDEGKSI	1.0451	−1.744
	PepO	KENYDGTDEFSLKNQK	1.288	−2.05
		SKLEVAPGYYVNGEIS	0.8605	−0.156
	6PGD	LKRKDDQGQDGPIVNY	1.4914	−1.531
		IGTGVSGGEKGALEGP	1.3547	−0.131
CTL	HP0197	DGKVVATNL	1.4692	0.222
	PepO	ALVPNLPIA	1.5535	1.467
	6PGD	KAGKKVSVY	1.3846	−0.444
HTL	HP0197	NDFKYQIKVENYIRQ	0.8205	−1.327
	PepO	EVKALVPNLPIAELV	0.6247	0.967
	6PGD	DSYLIEITADILKRK	0.5099	−0.18

## Data Availability

Data is available on request due to privacy restrictions.

## Supplementary Materials

The following supporting information can be downloaded at: https://www.mdpi.com/article/10.3390/vetsci10060383/s1, Table S1: The identification and evaluation of BCL epitopes; Table S2: The identification and evaluation of CTL epitopes; Table S3: The identification and evaluation of HTL epitopes; Figure S1: ProSA-web evaluation of the vaccine structure; Figure S2: ERRAT error prediction; Figure S3: Residues of the interaction between docked MVHP6 and TLR2; Figure S4: Residues of the interaction between docked MVHP6 and MHC I; Figure S5: Residues of the interaction between docked MVHP6 and MHC II.
