# Supplementary material for: Development of a Universal Multi-Epitope Vaccine Candidate against Streptococcus suis Infections Using Immunoinformatics Approaches
Source: Vet Sci. 2023 May 31;10(6):383. doi: 10.3390/vetsci10060383 (PMC10301797; doi:10.3390/vetsci10060383)
Supplement: Supplementary file 1 [file vetsci-10-00383-s001.zip › vetsci-2334676-supplementary.pdf]

**Table S1. The identification and evaluation of BCL epitopes.**

| Protein | Peptide | Position | Score | Antigenicity |
|---|---|---|---|---|
| HP0197 | MKKEEVKTTPSVQSNP | 494-509 | 0.85 | 1.1755 |
| | EEEYNAHLKQDEGKSI | 153-168 | 0.91 | 1.0451 |
| | PSVQSNPTLSHKGAPS | 503-518 | 0.87 | 1.0259 |
| | PAPIPDAPTPKVEEET | 277-292 | 0.93 | 1.0125 |
| | KAEDSAPKTAVPEVAP | 392-407 | 0.86 | 0.9703 |
| | APDAAPAPAPTPEVDP | 231-246 | 0.89 | 0.8808 |
| | PGDNTIEEEYNAHLKQ | 147-162 | 0.88 | 0.8736 |
| PepO | KENYDGTDEFSLKNQK | 161-176 | 0.89 | 1.288 |
| | SKLEVAPGYYVNGEIS | 538-553 | 0.85 | 0.8605 |
| | LKRKDDQGQDGPIVNY | 240-255 | 0.85 | 1.4914 |
| 6PGD | IGTGVSGGEKGALEGP | 123-138 | 0.89 | 1.3547 |
| | MKKEEVKTTPSVQSNP | 969-984 | 0.85 | 1.1755 |
| | EEEYNAHLKQDEGKSI | 628-643 | 0.91 | 1.0451 |
| | PSVQSNPTLSHKGAPS | 978-993 | 0.87 | 1.0259 |
| | PAPIPDAPTPKVEEET | 752-767 | 0.93 | 1.0125 |
| | KAEDSAPKTAVPEVAP | 867-882 | 0.86 | 0.9703 |
| | AQLRVASKENNWNLPF | 342-357 | 0.85 | 0.9185 |
| | APDAAPAPAPTPEVDP | 706-721 | 0.89 | 0.8808 |
| | PGDNTIEEEYNAHLKQ | 622-637 | 0.88 | 0.8736 |

**Table S2. The identification and evaluation of CTL epitopes.**

| Protein | Peptide | Position | Allele | Antigenicity | Immunogenicity |
|---|---|---|---|---|---|
| HP0197 | KERKVEEAL | 420-428 | HLA-B*40:01 | 0.9149 | 0.30758 |
| | KAGKKVSVY | 452-460 | HLA-A*30:02 | 1.3846 | 0.30454 |
| | DGKVVATNL | 466-474 | HLA-B*51:01 | 1.4692 | 0.44956 |
| PepO | LVKGSLEMK | 487-495 | HLA-A*68:01 | 0.9083 | 0.30154 |
| | ALVPNLPIA | 237-245 | HLA-A*02:01 | 1.5535 | 0.37921 |
| 6PGD | KERKVEEAL | 895-903 | HLA-B*40:01 | 0.9149 | 0.30758 |
| | KAGKKVSVY | 927-935 | HLA-A*30:02 | 1.3846 | 0.30454 |
| | QAGVPVPTF | 417-425 | HLA-A*32:01 | 1.0979 | 0.32054 |
| | ESRGYSVAI | 23-31 | HLA-B*51:01 | 1.3704 | 0.40123 |

**Table S3. The identification and evaluation of HTL epitopes.**

| Protein | Peptide | Position | Antigenicity | IL4 | IL10 | IFNγ |
|---|---|---|---|---|---|---|
| HP0197 | ANKKFRYSIRKFKVG | 4-18 | 0.8611 | inducer | inducer | positive |
| | NDFKYQIKVENYIRQ | 124-138 | 0.8205 | inducer | inducer | positive |
| | IRKFKVGVGSVLIAT | 12-26 | 0.4895 | inducer | inducer | positive |
| PepO | VQEIKATYAERIKAV | 359-373 | 0.7047 | inducer | non-inducer | negative |
| | EVKALVPNLPIAELV | 234-248 | 0.6247 | inducer | inducer | positive |
| | KMRALKKLDTLVVKI | 381-395 | 0.4739 | inducer | non-inducer | negative |
| | IVNYIMDAAGNKGTG | 252-266 | 1.0812 | inducer | non-inducer | negative |
| 6PGD | GVVGMAVMGRNLALN | 7-21 | 0.6756 | inducer | non-inducer | positive |
| | LLDEYFLDVTAKYQQ | 392-406 | 0.6437 | inducer | non-inducer | positive |
| | DSYLIEITADILKRK | 229-243 | 0.5099 | inducer | inducer | positive |
| | WRAGCIIRARFLQKI | 364-378 | 0.4151 | inducer | inducer | positive |

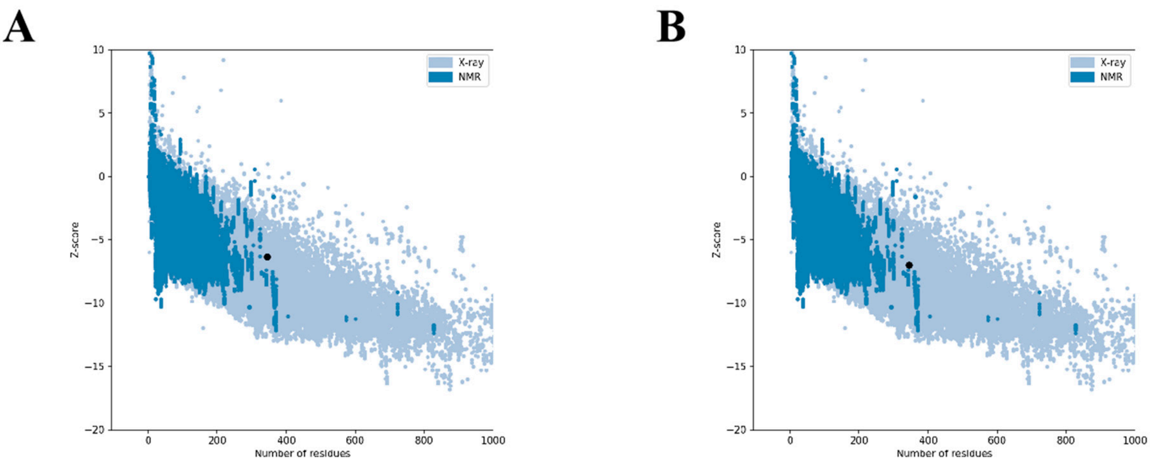


**Figure S1. ProSA-web evaluation of the vaccine structure.** ProSA-web results of the vaccine 3D structure before(A) and after (B) refined by GalaxyRefine. The value of Z-score was –6.36 and -7.01, respectively.

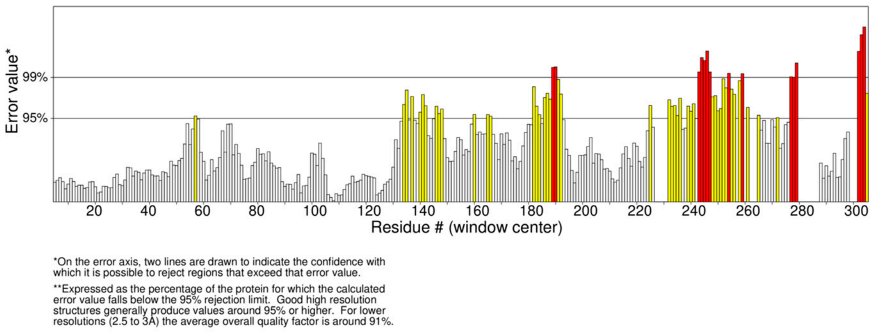


**Figure S2. ERRAT error prediction.** The quality of 3D model MVHP6 is 79.114.

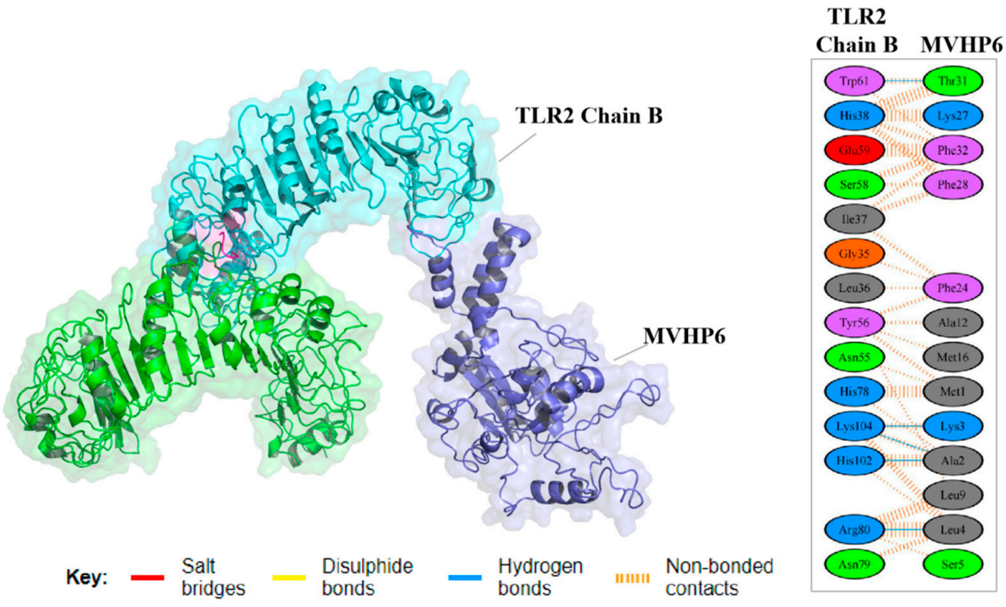


**Figure S3. Residues of the interaction between docked MVHP6 and TLR2.**

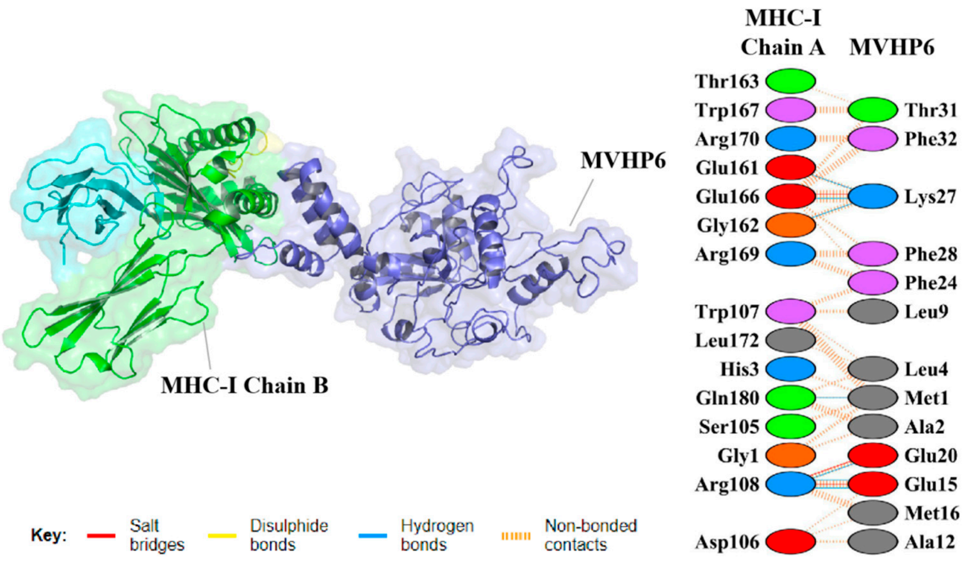


**Figure S4. Residues of the interaction between docked MVHP6 and MHC I.**

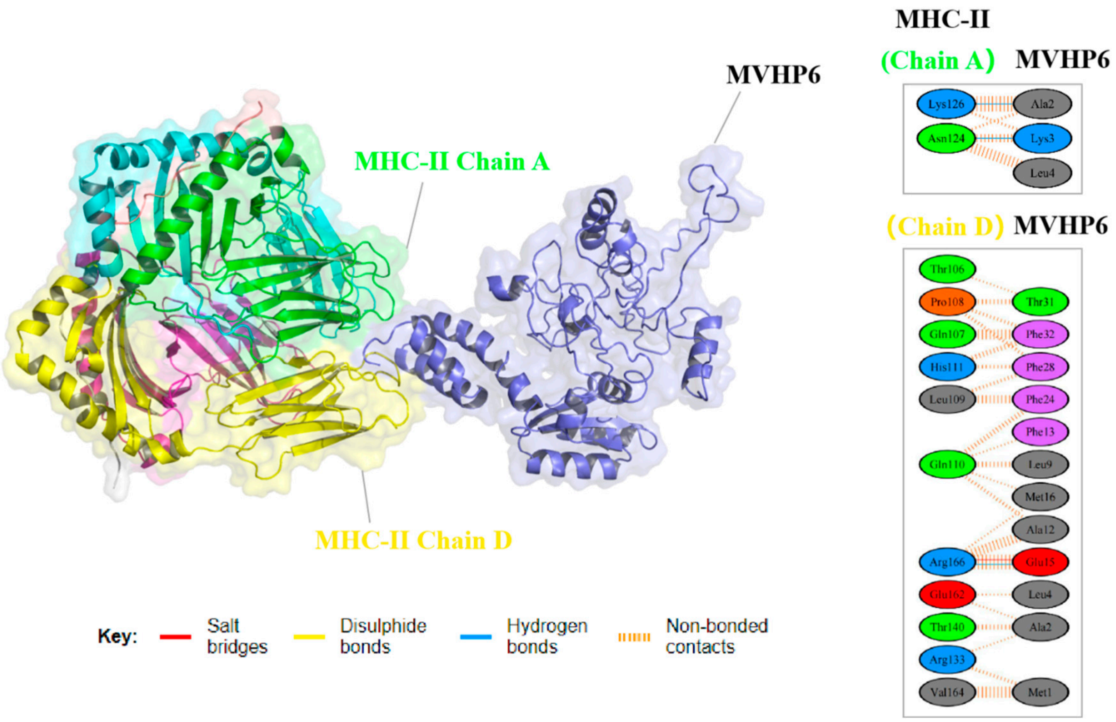


**Figure S5. Residues of the interaction between docked MVHP6 and MHC II.**
